# Senescence in Mesenchymal Stem Cells: Functional Alterations, Molecular Mechanisms, and Rejuvenation Strategies

**DOI:** 10.3389/fcell.2020.00258

**Published:** 2020-05-05

**Authors:** Jing Liu, Yue Ding, Zhongmin Liu, Xiaoting Liang

**Affiliations:** ^1^Research Center for Translational Medicine, Shanghai East Hospital, School of Medicine, Tongji University, Shanghai, China; ^2^Department of Cardiovascular Surgery, Shanghai East Hospital, Tongji University School of Medicine, Shanghai, China; ^3^Department of Organ Transplantation, Changzheng Hospital, Second Military Medical University, Shanghai, China; ^4^Institute for Regenerative Medicine, Shanghai East Hospital, School of Life Sciences and Technology, Tongji University, Shanghai, China

**Keywords:** aging, mesenchymal stem cells, senescence, mechanism, rejuvenation

## Abstract

Mesenchymal stem cells (MSCs) are multipotent cells capable of self-renewal and differentiation. There is increasing evidence of the therapeutic value of MSCs in various clinical situations, however, these cells gradually lose their regenerative potential with age, with a concomitant increase in cellular dysfunction. Stem cell aging and replicative exhaustion are considered as hallmarks of aging and functional attrition in organisms. MSCs do not proliferate infinitely but undergo only a limited number of population doublings before becoming senescent. This greatly hinders their clinical application, given that cultures must be expanded to obtain a sufficient number of cells for cell-based therapy. Here, we review the current knowledge of the phenotypic and functional characteristics of senescent MSCs, molecular mechanisms underlying MSCs aging, and strategies to rejuvenate senescent MSCs, which can broaden their range of therapeutic applications.

## Introduction

Mesenchymal stem cells (MSCs) were originally isolated from bone marrow (Friedenstein et al., 1968) but have since been detected in many tissues including dental pulp (Gronthos et al., 2002), adipose tissue (Zuk et al., 2002), and umbilical cord blood (Wang et al., 2004). The essential features of this heterogeneous cell population as defined by the International Society for Cellular Therapy (ISCT) in 2006 are adherence to plastic under culture conditions; expression of the cell surface markers CD44, CD90, CD105, and CD73; absence of the hematopoietic markers CD45, CD34, CD14, CD11b, CD79α, CD19, and human leukocyte antigen-DR; and multi-differentiation potential, with the capacity to generate osteoblasts, chondroblasts, and adipocytes (Dominici et al., 2006). According to the recently published ISCT position statement, although the classic set of markers still applies to *in vitro*-expanded MSCs, surface markers are evolving (Viswanathan et al., 2019). For example, while the definition of MSCs includes CD34 negativity, MSCs can be positive for this marker *in vivo* (Bellagamba et al., 2018). MSCs can differentiate into cells of ectodermal and endodermal parentage (Al-Nbaheen et al., 2013) and novel surface markers (CD165, CD276, and CD82) have been identified (Shammaa et al., 2020). Moreover, surface marker expression can change under certain culture conditions or when stimulated by a molecule (i.e., interferon-γ) (Stagg et al., 2006). Stringent functional criteria must be met for the designation of a cell as a “stem” cell (Viswanathan et al., 2019; Nolta et al., 2020). MSCs can be safely transplanted autologously or allogeneically as they have low immunogenicity, and thus have many potential applications in cell-based therapy for various disease states (Squillaro et al., 2016). To be clinically useful, MSCs must be expanded *in vitro* over several population doublings (PDs) to obtain a sufficient number of cells for immediate administration. The age of donors is a major factor determining the lifespan and quality of MSCs (Sethe et al., 2006; Baker et al., 2015); cells from aged donors perform less well than those from young donors because of their reduced proliferative capacity and differentiation potential. For patients with age-related diseases, allogeneic MSCs from healthy young donors are clearly preferable to autologous MSCs. On the other hand, regardless of donor age or whether the cells are autologous or allogeneic, MSCs inevitably acquire a senescent phenotype after prolonged *in vitro* expansion (Dimmeler and Leri, 2008; Li et al., 2017). *In vivo* aging refers to donor age, which affects the lifespan of MSCs; *in vitro* aging is the loss of stem cell characteristics by MSCs as they enter senescence during expansion in culture; and senescence is a state where cells stop dividing, which negatively affects their immunomodulatory and differentiation capacities, leading to reduced efficacy following administration (Fan et al., 2010; Turinetto et al., 2016). Thus, for MSCs to be clinically effective, it is essential to monitor senescence and understand the molecular basis of MSC aging. In this review, we discuss changes that occur in senescent MSCs, current strategies for monitoring senescence and the molecular mechanisms involved, and interventions that can potentially slow or even reverse this process.

## Current Status of MSC-Based Therapy

**FIGURE 1:**
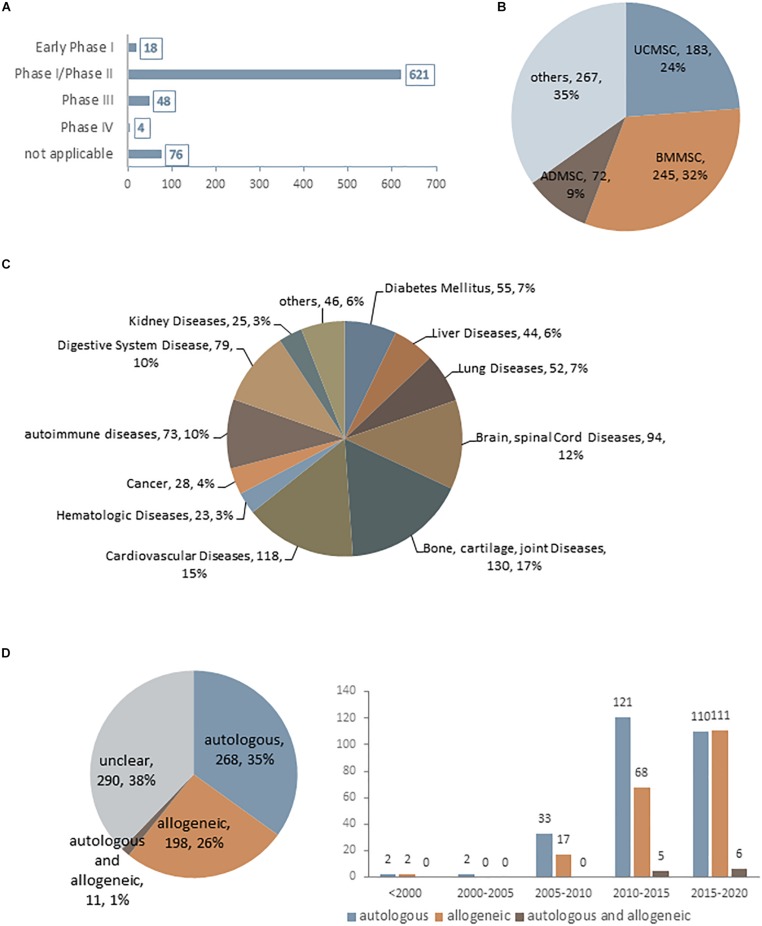
Current statistical data for MSC-based clinical trials as of January 2020 (data accessed from ClinicalTrials.gov ∼2020.1). **(A–D)** Statistics for MSC-based clinical trials in different phases **(A)**, using difference cell sources **(B)**, in different disease states **(C)**, and using autologous or allogeneic transplantation **(D)**.

Mesenchymal stem cells were first used therapeutically in human patients in 1995 (Galipeau and Sensebe, 2018) and has since been applied to the treatment of a broad spectrum of diseases. As of January 2020, there were 767 MSC-based trials registered at www.ClinicalTrials.gov, most of which are at an early phase (phase I or I/II) (Figure 1A). Although MSCs have been obtained from a variety of human sources, those derived from bone marrow, umbilical cord, and adipose tissue are preferred for clinical applications and account for approximately 65% of MSCs being used (Figure 1B). Due to their multi-differentiation potential and immunomodulatory and paracrine effects, MSCs have been extensively applied in various diseases (Figure 1C). Interestingly, although autologous transplantation was initially favored over allogeneic MSCs, there has been a notable increase in the use of the latter over the past decade (Figure 1D); for example, 11 out of 19 industry-sponsored phase III clinical trials of MSCs used allogeneic transplantation (Wang et al., 2016; Galipeau and Sensebe, 2018). One reason for this popularity is their low immunogenicity—that is, allogeneic MSCs can be safely transplanted without a high risk of rejection by the recipient (Wang D. et al., 2013; Lee et al., 2016). Additionally, candidate patients for cell-based therapy usually have age-related diseases. While the regenerative capacity of MSCs declines markedly with age (Kretlow et al., 2008; Yu et al., 2011), autologous transplantation is not the best option for these patients. However, robust immunologic data from clinical trials using allogeneic MSCs are still lacking. Although MSCs are considered as immunoprivileged, their transdifferentiation into other cell types—a basic property of MSCs–can increase the risk of immunogenicity (Mukonoweshuro et al., 2014; Ryan et al., 2014). Thus, there is still much to learn and optimize in terms of *in vivo* MSC interactions in pathologic states, which can lead to a better understanding of MSC aging and improve the long-term safety and outcome of MSC engraftment.

### Features of MSC Senescence

**FIGURE 2:**
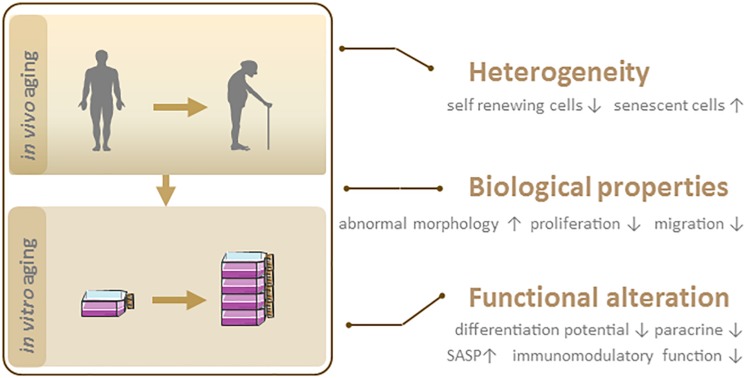
Phenotypic features of senescent MSCs. *In vivo* and *in vitro* aging lead to MSC senescence, which is characterized by heterogeneity, biological and functional changes.

Irrespective of their source, MSCs enter a state of replicative senescence (i.e., *in vitro* aging, also known as the Hayflick limit) after repeated serial passage in culture when the cells stop dividing after a certain number of PDs (Hayflick and Moorhead, 1961). The maximum number of PDs that can be achieved by MSCs is estimated to be 30 to 40 (Banfi et al., 2002; Baxter et al., 2004). No clear information on passage number has been provided for the 15 MSC products approved to date for clinical use (Table 1). However, given that the differentiation potential of MSCs decreases after extended passages, low-passage cultures are recommended for clinical-scale expansion of cultures (Lechanteur et al., 2016). In the following sections, we discuss heterogeneity and biological and functional changes in MSC senescence (Figure 2).

**TABLE 1:** Approved MSC-based medicinal products.

**Product name**	**Time of approval**	**MSC source**	**auto/allo**	**Country**	**Company**	**Disease**
Hearticellgram-AMI	2011-07-01	BMMSCs	auto	Korea	FCB-Pharmicell	Acute myocardial infarction
Cuepistem	2012-01-18	ADMSCs	auto	Korea	Anterogen	Crohn’s disease complicated with anal fistula
Cartistem	2012-01-19	UCBMSCs	allo	Korea	Medipost	Degenerative arthritis
Prochymal/remestemcel-L	2014-05-02	BMMSCs	allo	Canada	Mesoblastinterna-tionalsar	Pediatric acute graft versus host disease (aGvHD)
Neuronata-R	2014-07-30	BMMSCs	auto	Korea	Corestem	Lateral sclerosis of spinal cord
Temcell HS	2015-09-20	BMMSCs	allo	Japan	JCR Pharmaceuticals	Acute graft versus host disease (aGvHD)
Stempeucel	2016-03	BMMSCs	allo	India	Stempeutics Research	Severe limb ischemia caused by thromboangiitis obliterans (Buerger disease)
Alofisel (darvadstrocel, Cx601)	2018-03-27	ADMSCs	allo	Japan and Belgium	Takeda Pharmaceutical Company and TiGenix NV	Complex perianal fistulas in Crohn’s disease
Holoclar	2015-02-17	Limbal stem cells	auto	Italy	Chiesi Farmaceutici S.p.A	Restoration of Corneal Epithelium in Patients With Limbal Stem Cell Deficiency
MPC	2010-07	Mesenchymal precursor cell	auto	Australia	Mesoblast	Fracture healing and disc healing
ChondroCelect	2009-10-05	Cartilage cells	auto	Belgium	TiGenix NV	Osteoarthritis of the knee and repair cartilage damage of femoral condyle in adult knee joint.
Prochymal	2009-12	BMMSCs	allo	United States	Osiris Therapeutics	Diabetes mellitus type I
MultiStem	2012-07	BMMSCs	allo	United States	Athersys	Hurler’s syndrome/ischemic stroke
Maci	2016-12	Cartilage cells	auto	United States	–	Osteochondral damages
Hemacord	2011-11	UCBMSCs	allo	United States	New York Blood Center	Hemorrhagic disease

*Auto, autologous; allo, allogeneic; BMMSCs, bone marrow-derived mesenchymal stem cells; ADMSCs, adipose-derived mesenchymal stem cells; UCBMSCs, umbilical cord blood-derived mesenchymal stem cells.*

### Phenotypic Heterogeneity of Senescent MSCs

**FIGURE 3:**
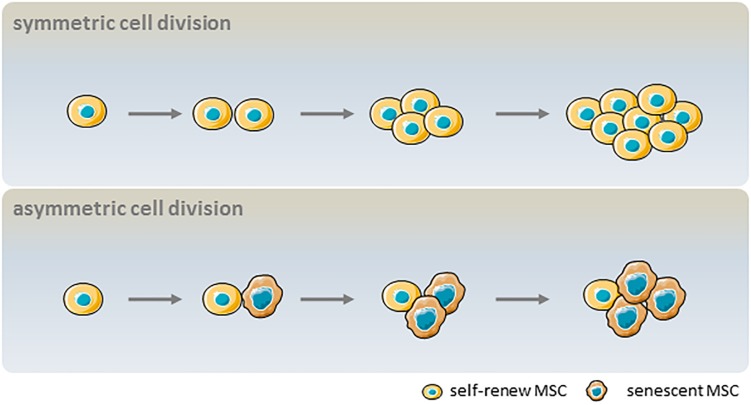
Influence of asymmetric cell division kinetics on the heterogeneity of MSC senescence. In symmetric cell division, a parent MSC with self-renewal capacity divides into two daughter MSCs that can also self-renew. In asymmetric cell division, a parent MSC with self-renewal capacity divides into one self-renewing and one non-dividing cell that becomes senescent in culture.

Despite their global features as defined by the ICST, MSCs are complex cell populations that exhibit heterogeneity depending on the donor, tissue source, and whether they are clonal populations or single cells (Phinney, 2012). MSC heterogeneity comprises proliferation rate, morphology, immunophenotype, multilineage differentiation potential, and senescence (Schellenberg et al., 2012). In symmetric cell division, a self-renewing parent cell divides into two daughter cells with comparable shape and differentiation potential. In contrast, asymmetric cell division yields a self-renewing cell and a non-dividing cell that becomes senescent in culture. These dynamics result in an initially dominant cell population being overtaken by other clonal populations after multiple passages (Figure 3). Heterogeneity in the proliferation potential of cultured MSCs manifests morphologically as subpopulations of small, round, rapidly proliferating cells and slowly dividing, large flattened cells (Mets and Verdonk, 1981; Colter et al., 2001). Using the limiting dilution assay at later passages, it was determined that not every cell is capable of clonal expansion and colony formation at the time of culture establishment (Schellenberg et al., 2012). More importantly, the number of colony-forming unit (CFU) fibroblasts decreased continuously during culture expansion, and were scarcely detected after >20 passages (Schellenberg et al., 2012). Likewise, clonal analysis of single-cell–derived colonies has suggested that not every cell has trilineage (i.e., osteogenic, adipogenic, and chondrogenic) potential (Wagner et al., 2008), and subsets with high differentiation potential rapidly decline in number after a few passages (Schellenberg et al., 2012). The age-related heterogeneity of MSCs is thought to be associated with epigenetic status; subpopulations with variable expression of stem cell antigen (Sca)-1 regained the Sca-1 profile of the parent cell after 4–8 days of culture, which was accompanied by epigenetic changes at the lymphocyte antigen 6 complex promoter (Hamidouche et al., 2017). Given the relevance to clinical efficacy, molecular markers of aging in cultured MSCs are needed to reflect senescence-associated alterations. The current understanding of aging-related cellular changes is based primarily on homogeneous bulk cell-derived data; emerging tools for single-cell analysis can help to define the heterogeneity of MSCs.

As MSC populations with a large proportion of senescent cells are less effective when transplanted, it is critical to detect senescent MSCs during expansion. Surface marker profiling is one approach for identifying and purifying senescent cells from a culture. For example, the expression of CD146—also known as melanoma cell adhesion molecule (MCAM)–was downregulated in MSCs derived from aged donors compared to those from young donors, as well as in MSCs after prolonged *in vitro* expansion (Gnani et al., 2019). Furthermore, low but not high CD146 expression was associated with a senescent phenotype in MSCs (Jin et al., 2016), and CD146^+^ MSCs showed increased migratory potential toward degenerating tissues (Wangler et al., 2019). CD264 is another surface marker of *in vitro* aging in MSCs that is unrelated to the chronologic age of the donor (Madsen et al., 2017); cells expressing this protein exhibit increased senescence-associated β-galactosidase (SA-β-gal) activity and reduced differentiation potential and colony-forming efficiency compared to CD264^–^ MSCs (Madsen et al., 2020). Other surface markers that show altered expression with *in vitro*/*in vivo* aging are summarized in Table 2. It should be noted that although these molecules are expressed to varying degrees during the aging process and may be associated with functional changes, there is presently no consensus on whether they can serve as the gold standard for selective purification of young vs. old MSC populations.

**TABLE 2:** Surface marker alteration in senescent MSCs.

**Surface marker**	***In vivo* aging/*In vitro* aging**	**Type of MSCs**	**PMID**
**Decreased expression in senescent MSCs**			
CD146/MCAM	*in vitro* aging	Human-BMMSCs	29751774
	*in vitro* aging	Human periapical cyst derived MSCs	27406247
	*in vitro* aging	Human tonsil derived MSCs	25155898
	*in vitro* aging	Human-UCBMSCs	26941359
	*in vitro* aging	Human-UCBMSCs	21144825
CD106/VCAM-1	*in vitro* aging	Human-BMMSCs	29751774
	*in vitro* aging	Human-BMMSCs	30211967
	*in vitro* aging	Human-UCBMSCs	21144825
	*in vitro* aging	Human vertebral body spongiosa derived MSCs	19242838
CD90	*in vitro* aging	Human-AFMSCs	27803714
	*in vitro* aging	Human-UCBMSCs	21144825
CD105	*in vitro* aging	Human-AFMSCs	27803714
	*in vitro* aging	Human-UCBMSCs	21144825
CD44	*in vitro* aging	Human-AFMSCs	27803714
	*in vitro* aging	Human-UCBMSCs	21144825
CD49F	*in vitro* aging	Human-BMMSCs	26013602
CD34	*in vivo* aging	Human-BMMSCs	23197850
CD133	*in vivo* aging	Human-BMMSCs	23197850
CD166	*in vitro* aging	Human-UCBMSCs	21144825
**Increased expression in senescent MSCs**			
CD264	*in vivo* aging	Human-BMMSCs	28962588
	in vivo aging	Human-BMMSCs	31612990
HLA/MHC	*in vitro* aging	Human-BMMSCs	30211967
	*in vitro* aging	Human-ADMSCs	22391697
CD49C	*in vitro* aging	Human-BMMSCs	30211967
CD45	*in vitro* aging	Human-ADMSCs	22391697
**Controversial**			
CD271/P75NTR	*in vivo* aging	Human-BMMSCs	23197850
	*in vivo* aging	Human-BMMSCs	31467563

*BMMSCs, bone marrow-derived mesenchymal stem cells; UCBMSCs, umbilical cord blood-derived mesenchymal stem cells; AFMSCs, amniotic fluid derived-mesenchymal stem cells; ADMSCs, adipose-derived mesenchymal stem cells.*

### Biological Properties of Senescent MSCs

Early-passage MSCs are small and have a fibroblast-like spindle shape but acquire a hypertrophic and flat morphology with more podia and actin stress fibers upon extended culture (Stenderup et al., 2003; Mauney et al., 2004; Stolzing and Scutt, 2006). MSCs from passages 1 to 3 have a uniform size but begin to enlarge at passage 5, such that cells at passages 6–9 are 4.8-fold larger than passage 1 cells (Oja et al., 2018). Morphologic features can predict how well cells can adapt to a given condition. Cell and nuclear morphology in MSCs in the first 3 days of osteogenic induction was found to be closely correlated with their long-term (35-day) mineralization capacity (Marklein et al., 2016). A subset of aged MSCs with a small cell size had ATP levels equivalent to those in young MSCs, whereas levels in large-sized cells were comparable to those in the aged parent MSC population (Block et al., 2017). Increased cell size and granularity were positively correlated with MSC autofluorescence; the latter has therefore been proposed as a non-invasive, real-time quantifiable marker for cellular senescence (Bertolo et al., 2019).

Animal cells sense and correct deviations in size by adjusting cell cycle length as well as growth rate (Miettinen et al., 2014), which is increased in small cells and reduced in large cells (Ginzberg et al., 2018). Loss of cell size uniformity can indicate abnormal biosynthesis or cell cycle progression. While the regulation of size homeostasis in relation to senescence is not fully understood, cell enlargement can distinguish senescent subpopulations in culture. Among aged MSCs, SA-β-gal activity is increased in large as compared to small-sized cells (Block et al., 2017). Notably, MSCs immortalized by SV40 (Negishi et al., 2000) or telomerase transfection (Kobune et al., 2003) are significantly smaller than their parent cells.

The enlargement of aging cells and their transformation to a hypertrophic morphology is accompanied by biological changes. A decline in proliferative capacity was reported in MSCs derived from old patients as compared to their healthy young counterparts (Banfi et al., 2002) and in long-term MSC cultures regardless of the cell source. MSCs from young donors had greater mitotic activity (41 ± 10 vs. 24 ± 11 PDs), slower progression to senescence, and an increased rate of proliferation (0.09 ± 0.02 vs. 0.05 ± 0.02 PDs/day) than those from old donors (Stenderup et al., 2003). CFU is a retrospective parameter describing the clonogenic potential of a single cell; decreases in CFU and average colony size are correlated with MSC aging *in vitro* (Liu et al., 2004).

In addition to impaired proliferation, aging negatively affects MSC migration and homing ability (Liu et al., 2017). Directed migration toward stimuli by MSCs is critical for better functional outcomes in cell-based therapy. Impaired migratory capacity in senescent MSCs in response to pro-inflammatory signals was found to be closely associated with activator protein (AP)-1 pathway inhibition (Sepulveda et al., 2014). Cell migration involves the reorganization of the actin cytoskeleton (Le Clainche and Carlier, 2008). MSCs derived from old donors exhibit reduced response to biological and mechanical signals because their actin cytoskeleton is less dynamic (Kasper et al., 2009). Gene expression profiling has identified several cytokines and chemokines and their receptors important for cell migration–including stromal cell-derived factor 1 (SDF-1) and its receptor chemokine receptor type 4 (CXCR4), tumor necrosis factor receptor (TNFR), IFN-γ receptor (IFNGR), and C-C motif chemokine receptor 7 (CCR7)—that are downregulated in aged MSCs as compared to younger cells (Geissler et al., 2012; Bustos et al., 2014).

### Functional Changes Associated With MSC Senescence

A basic strategy for MSC-based regeneration is to replace cells that are lost or impaired by disease with functional cells. It was previously thought that MSCs exert their therapeutic effect through *trans*-differentiation. During *in vitro* culture, MSCs progressively lose their capacity to differentiate into adipogenic and osteogenic lineages although the preferred fate is debated, with some studies suggesting that aging shifts the balance in favor of adipocytes at the expense of osteoblastogenesis (Stolzing et al., 2008), and others reporting that osteogenic activity is preserved or even increased in late passages (Wagner et al., 2008) or that both osteogenic and adipogenic potential is lost (Geissler et al., 2012). Age-associated changes in differentiation potential may be related to altered susceptibility to reactive oxidative species (ROS) and apoptosis (Bruedigam et al., 2010); MSCs undergoing osteoblast differentiation showed dose-dependent increases in apoptosis and ROS accumulation upon treatment with rosiglitazone, whereas adipogenesis was unaffected (Jiang et al., 2008). Lineage bias in differentiation is regulated by key signaling pathways, intracellular oxidative stress, and transcriptional and post-transcriptional mechanisms. Gene expression analysis has revealed an age-related downregulation of osteoblast transcription factors such as core binding factor α1 (CBFA1), runt-related transcription factor 2 (Runx2), and distal-less homeobox 5 (DIx5) as well as collagen and osteocalcin, and upregulation of adipogenic factors such as peroxisome proliferator-activated receptor-γ (PPAR-γ) and adipocyte fatty acid-binding protein (aP2) (Jiang et al., 2008). The target genes activated by PPAR-γ are related to lipid metabolism and adipocyte differentiation. Thus, the age-related increase in PPAR-γ expression shifts the fate of MSCs toward adipogenesis, and Wnt/β-catenin signaling regulates MSC differentiation by suppressing PPAR-γ and biasing differentiation toward osteoblastogenesis (Xu et al., 2016).

Aging cells acquire a senescence-associated secretory phenotype (SASP) involving the secretion of proteins that can affect the behavior of neighboring cells via autocrine/paracrine mechanisms (Borodkina et al., 2018; Campisi et al., 2019). MSCs have potent anti-inflammatory and immunosuppressive functions and thus have therapeutic potential for inflammation-related diseases. Aged MSCs have a diminished capacity for inhibiting the proliferation of allogeneic peripheral blood mononuclear cells compared to younger cells (Gnani et al., 2019). The activation of SASP factors such as interleukin 6 (IL-6), IL-8, and monocyte chemotactic protein 1 (MCP1) in the conditioned medium of aged MSCs was shown to be increased compared to young MSC cultures at early passages, an effect that was exacerbated at late passages (Gnani et al., 2019). Secretion of SASP-related chemokines/cytokines not only drives responses that reinforce senescence in a cell-autonomous manner but also acts on neighboring cells via a paracrine mechanism to accelerate senescence. For example, factors secreted by aged MSCs were shown to activate pro-inflammatory gene expression in young hematopoietic stem cells and decreased their clonogenic potential (Gnani et al., 2019).

The beneficial effects of MSC-based therapy are attributable to the action of pro-angiogenic paracrine factors. In aged MSCs, the secretion of these factors—including vascular endothelial growth factor (VEGF), placental growth factor (PGF), and hepatic growth factor (HGF)—is reduced, whereas that of anti-angiogenic factors such as thrombospondin-1 (TBS1) and plasminogen activator inhibitor-1 (PAI-1) is increased. Thus, age negatively affects angiogenesis and directly undermines the therapeutic efficacy of MSCs (Efimenko et al., 2011; Khan et al., 2011).

### Strategies for Monitoring MSC Senescence

β-D-Galactosidase (β-Gal) is a eukaryotic hydrolase localized in the lysosome that is active at the optimal pH (6.0) in senescent cells but is absent in proliferating cells (Dimri et al., 1995). SA-β-gal activity is suggested as the gold standard for evaluating senescence in cells and can be detected by cytochemistry/histochemistry and fluorescence-based methods. However, when used in combination with other markers, it can yield false-positive/negative results in quiescent cells or upon stress (de Magalhaes et al., 2004; Yang and Hu, 2005). Senescence-associated lysosomal α-L-fucosidase (SA-α-Fuc) has recently been identified as a more robust biomarker in all types of cellular senescence (Hildebrand et al., 2013; Singh and Piekorz, 2013), but there is still limited evidence for its sensitivity and specificity in distinguishing senescent MSCs.

Telomeres are specialized nucleoprotein caps containing repetitive nucleotide sequences that protect chromosomes from end-to-end fusion and prevent the loss of genetic information during DNA replication (Sahin and Depinho, 2010). Telomeres shorten with every cell division and senescence is triggered when they reach a critical length (Baird et al., 2003). As such, telomere length has been used to estimate replicative history and predict senescence in MSCs (Montpetit et al., 2014). There is increasing evidence of an association between diminished proliferative capacity and telomere shortening in MSCs. However, the exact telomere length in senescent MSCs and whether it differs according to cell source, culture conditions, and measurement method is unclear. For example, a telomere length of 10 kb was proposed as a threshold for senescence (Baxter et al., 2004), although another study reported a length of 6.8 ± 0.6 kb in senescent cells (Oja et al., 2018). In addition, a recent study described a mechanism of senescence that is independent of cell division and telomere length, involving activation of classical senescence-associated pathways and yielding a non-canonical SASP (Anderson et al., 2019). Although this phenomenon was first reported in post-mitotic cardiomyocytes, it may also occur in MSCs. Thus, telomere shortening has limitations for the measurement of senescence, and other markers may be more informative under certain conditions.

The senescent state is characterized by cell cycle arrest. Senescence-associated growth arrest is maintained by the activation of several pathways including phosphorylated inhibitor of cyclin-dependent kinase 4A (p16^*INK4A*^)/phosphorylated retinoblastoma (pRb) and p53/p21^*WAF1*^ signaling (Campisi, 2005). p16^*INK4A*^ is an inhibitor of cyclin-dependent kinase (CDK) and induces premature cell senescence via telomere-dependent and -independent mechanisms (Serrano et al., 1993). p16^*INK4A*^ level was shown to increase with chronological age or PDs of MSCs in culture, and a large proportion of the p16^*INK4A*^-positive cells were negative for the proliferation marker Ki67 and positive for SA-β-gal. Inhibiting p16^*INK4A*^ reduced the number of senescent MSCs and conferred cells with the ability to proliferate (Shibata et al., 2007). Similarly, overexpressing p21^*WAF1*^—a CDK inhibitor that acts by dephosphorylating pRb–increases cellular senescence, as evidenced by elevated SA-β-gal activity and telomere shortening (Huang et al., 2004), while inhibiting p21^*WAF1*^ in senescent cells restored their replicative capacity. However, p21^*WAF1*^ depletion was less efficient at preventing senescence than p53 depletion, suggesting that the latter acts through p21^*WAF1*^-independent mechanisms to exert this effect (Gire and Dulic, 2015).

Mesenchymal stem cells-derived microvesicles (MSC-MVs) that mimic the senescent state of the parent MSC have recently emerged as a potential cell-free biomarker for cellular senescence. Senescent late-passage MSCs secrete larger amounts of MSC-MVs of smaller size than those in early passages, and CD105 expression in MSC-MVs decreased with senescence in parent MSCs. RNA sequencing results suggest that most genes that are highly expressed in senescent MSC-MVs are involved in aging-related diseases (Lei et al., 2017). Functionally, senescent MSC-MVs have a lower capacity to promote osteogenesis (Lei et al., 2017) and recruit macrophages and fail to alter macrophage phenotypes (Huang et al., 2019).

Besides the abovementioned markers, new tools and approaches have been proposed to monitor MSC aging such as SiR-actin, a fluorogenic F-actin specific probe that can be used to evaluate actin turnover (Mishra et al., 2019). CyBC9 (another fluorescent probe) combined with high-throughput screening revealed accumulation of mitochondria in senescent MSCs that presumably resulted from the loss of membrane potential (Ang et al., 2019). Thus, senescence can be characterized not by a universal biomarker, but by a set of non-exclusive markers in conjunction with specific biological features.

### Role of DNA Damage, ROS, and Autophagy in MSC Senescence

**FIGURE 4:**
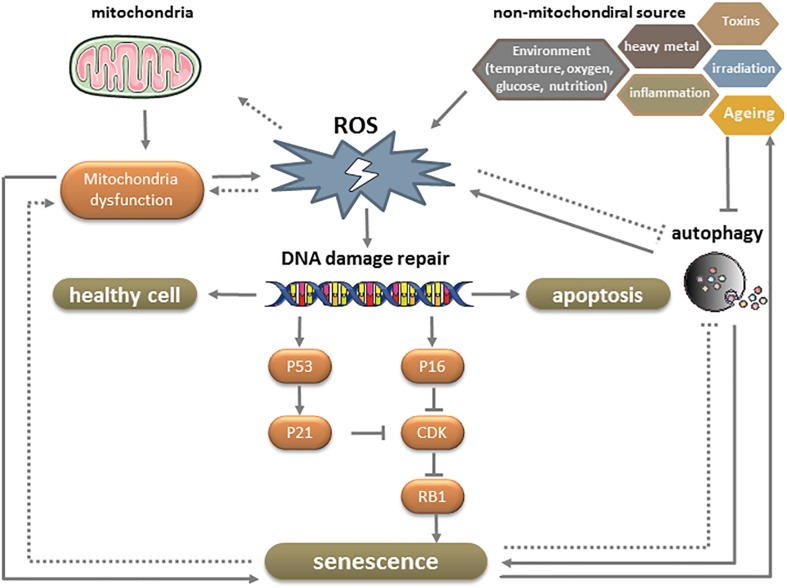
Effect of ROS on MSC senescence.

Senescence is a multistep process involving various mechanisms that have not been fully elucidated. One of these is irreversible cell cycle arrest—typically in response to DNA damage—in the presence of growth-promoting stimuli. DNA damage accumulates throughout the lifetime of an organism as a result of DNA replication errors and exposure to endogenous and exogenous mutagens. The DNA damage response (DDR) network can sense and initiate repair of mutations and thereby slow their accumulation. Activation of the DDR network can transiently halt cell cycle progression through stabilization of p53 and transcriptional activation of the CDK inhibitor p21. However, if DNA damage persists, p16^*INK4A*^ is activated via p38-mitogen-activated protein kinase-mediated mitochondrial dysfunction and ROS production. This results in CDK inhibition and activation of the tumor suppressor Rb1, which induces the onset of senescence (Figure 4; Zhang et al., 2007; Ciccia and Elledge, 2010).

Reactive oxidative species are a group of oxygen-containing small molecules. Approximately 90% of ROS are generated endogenously by the mitochondrial electron transport machinery (Poyton et al., 2009) but extrinsic factors such as radiation, ultraviolet light, hypoxia, and low temperature can also increase their production. As a metabolic by-product, ROS induce oxidation and various cellular responses through the generation of reactive secondary metabolites. While physiologic levels of ROS are necessary for proliferation and differentiation, an excess can trigger cellular senescence, including in MSCs (Sart et al., 2015). ROS induce DNA damage and accelerates telomere erosion, both of which activate the DDR. Senescent MSCs have elevated levels of ROS compared to normal cells that cause persistent DDR activation, thereby forming a positive feedback loop in the progression of senescence (Figure 4; Yang et al., 2015).

Reactive oxidative species accumulate with advancing age, leading to decreased mitochondrial metabolism. Mitochondrial DNA (mtDNA) encodes 13 polypeptides of the mitochondrial oxidative phosphorylation (OXPHOS) enzyme complexes, 22 tRNAs for mitochondrial biosynthesis, and 16S and 18S rRNA for mitochondria. Because of the absence of efficient repair mechanisms, the mutation rate of mtDNA is higher than that of nuclear DNA. A point mutation in mitochondria-encoded subunit 3 of cytochrome c oxidase was found to be associated with enhanced tissue degeneration and risk of premature aging (Niemann et al., 2017). The mitochondrial free radical theory of aging posits that age-dependent accumulation of mtDNA abnormalities, impaired OXPHOS, and altered expression of antioxidant enzymes lead to increased ROS production, which, in turn, results in progressive mitochondrial dysfunction and global cellular damage in a positive feedback loop (Figure 4; Wang C. H. et al., 2013). However, other studies suggest that mtDNA mutations and mitochondrial dysfunction affect aging independently of ROS production, based on the unexpected observation that genetic manipulations that increased mitochondrial ROS levels and oxidative damage did not accelerate aging in mice (Zhang et al., 2009), whereas those that impaired mitochondrial function without increasing ROS enhanced aging (Kujoth et al., 2005).

Autophagy is a critical process for maintaining cellular homeostasis under physiologic and pathologic conditions. By removing damaged cellular components including proteins and mitochondria, autophagy prevents age-related cellular injury (Wirawan et al., 2012) and allows stem cells to avoid the transformation from a reversible quiescent (G0) state to irreversible senescence (Garcia-Prat et al., 2016). Although ROS are known to stimulate autophagy, the age-related increase in ROS levels reduces autophagic capacity (Yamamoto et al., 2016). In fact, aged MSCs exhibit reduced autophagy, which is correlated with diminished self-renewal capacity and regenerative potential and replicative exhaustion. From a mechanistic standpoint, decreased autophagy results in the loss of proteostasis and increases mitochondrial activity, oxidative stress, and metabolism state in MSCs (Figure 4; Revuelta and Matheu, 2017).

Key aspects of the mechanisms of MSC aging remain unknown, but epigenetic changes (i.e., those occurring in the absence of DNA sequence alterations) such as DNA methylation, histone modification, and chromatin remodeling may play a role (Ozkul and Galderisi, 2016; Cakouros and Gronthos, 2019). For example, dysregulated expression of Brahma-related gene 1 (BRG1), a component of ATP-dependent chromatin remodeling complexes, was associated with senescence in MSCs via regulation of NANOG methylation status (Squillaro et al., 2015). Additionally, microenvironmental and hormonal conditions are important factors contributing to MSC aging *in vivo*. As MSCs exist in a semi-static state, replicative exhaustion is unlikely to occur (Ganguly et al., 2017). There is increasing evidence that the *in vivo* cellular aging process is caused by chronologic aging of the host and is accelerated by conditions such as obesity and systemic inflammation (Frasca et al., 2017; Franceschi et al., 2018).

### Strategies for Rejuvenating Senescent MSCs

Strategies allowing the generation of large numbers of MSCs that have retained their stemness are needed for clinical applications. Here, we summarize current research efforts to prevent MSC senescence.

Induced pluripotent stem cell (iPSC)-derived MSCs (iMSCs) can be passaged more than 40 times without exhibiting features of senescence (Sabapathy and Kumar, 2016). iMSCs retain a donor-specific DNA methylation profile while tissue-specific, senescence-associated, and age-related patterns are erased during reprogramming (Frobel et al., 2014). Recent studies have demonstrated that iMSCs have superior regenerative capacity compared to tissue-derived MSCs in preclinical degenerative disease models (Lian et al., 2010; Chen et al., 2019; Wang et al., 2019). However, the generation of iMSCs from iPSCs requires a significant degree of molecular manipulation, and there are safety concerns regarding the self-renewal and pluripotency of iPSC-derived cells after *in vivo* transplantation, which have the risk of tumorigenicity and genomic instability (Hynes et al., 2013). In addition, each independent iPSC line has a unique genetic and epigenetic profile that must be characterized. The concept of iMSCs is at its infancy and requires validation from preclinical and clinical studies before it can be clinically useful.

Aging is not a passive or random process but can be modulated through several key signaling molecules/pathways (Kenyon, 2010). Identification of age-related coordinating centers can provide novel targets for therapeutic interventions. Sirtuins (SIRTs) are a class of highly conserved nicotinamide adenine dinucleotide-dependent protein deacylases of which there are 7 (SIRT1–7) in mammals (Vassilopoulos et al., 2011). The role of SIRTs in aging is related to their regulation of energy metabolism, cell death, and circadian rhythm and maintenance of cellular and mitochondrial protein homeostasis (O’Callaghan and Vassilopoulos, 2017). Mitochondrial SIRTs (SIRT3–5) act as stress sensors and regulate protein networks to coordinate the stress response (van de Ven et al., 2017). Overexpression of SIRTs has been investigated as a potential strategy for preventing MSC aging. For instance, SIRT3 expression in MSCs decreased with prolonged culture and its overexpression in later-passage cells restored differentiation capacity and reduced aging-related senescence (Denu, 2017). SIRT1 is required for long-term growth of MSCs and SIRT1 overexpression was shown to delay senescence without loss of adipogenic or osteogenic potential (Yuan et al., 2012). Additionally, SIRT1 expression was shown to be spontaneously upregulated upon osteogenic differentiation and protected MSCs from extracellular oxidative stress (Li et al., 2018).

Genetic engineering has been used to slow MSC aging. Besides SIRTs, several molecules have been identified as potential targets for interventions to prevent senescence (Table 3). Ectopic expression of telomerase reverse transcriptase in MSCs extended their replicative lifespan, which preserved a normal karyotype, promoted telomere elongation, and abolished senescence without loss of differentiation potential (Simonsen et al., 2002). Introduction of Erb-B2 receptor tyrosine kinase 4 (ERBB4) in aged MSCs conferred resistance to oxidative stress-induced cell death and rescued the senescence phenotype (Liang et al., 2019). Knocking down macrophage migration inhibitory factor (MIF) in young MSCs induced senescence; conversely, its overexpression in aged MSCs rejuvenated the cells by activating autophagy (Zhang et al., 2019). However, the risk of malignant transformation remains a major barrier for the use of genetics-based approaches in clinical practice.

**TABLE 3:** Strategies for MSC rejunvenation.

	**Interventing approach/medicine**	**Mechanism**	**Rejunvenation of function**	**Target cell**	**PMID**
Genetic	miR-195 inhibition	Induced telomere relengthening	Proliferation	Human-BMMSCs	26390028
approach		Reduced SA-β-gal expression			
		Restored antiaging factors expression including Tert and SIRT1			
		Restored phosphorylation of AKT and FOXO1			
	ERBB4 overexpression	Inhibited PI3K/AKT and MAPK/ERK pathways	Angiogenesis	Mouse-MSCs	25996292, 30566395
			Survival		
			Mobility		
			Apoptotic resistance		
	SIRT1 overexpression	Decreased H2O2-induced oxidative stress response capabilities	Senescent phenotype	Rat-MSCs	25034794, 28258519
		Increased Ang1, bFGF expressions, decreased TBS1 expressions	Angiogenesis		
		Increased in Bcl-2/Bax ratio	Apoptosis		
	SIRT3 overexpression	Reduced ROS	Senescent phenotype	Human-BMMSCs	28717408
			Adipocytes/osteoblasts differentiation		
	TERT overexpression	Increased telomere length, prolonged population doublings	Osteoblastic differentiation	Human-BMMSCs	12042863
			Proliferation		
	p16^*INK4A*^ Knockdown	Up-regulated TGF-β expression	Senescent phenotype	Human-BMMSCs	22820504
		Increased the percentage of Treg cells			
		Up-regulated ERK1/2 activation			
	p21 Knockdown	Increased the level of Cyclin E, cyclin-dependent kinase-2	Proliferation	Human-BMMSCs	24151513
		Increased the phosphorylation of retinoblastoma protein	Senescent phenotype		
	Silencing lincRNA-p21	Interacted with the WNT/β-catenin signaling pathway	Proliferation and paracrine function	Mouse-BMMSCs	28901439
	PTEN or p27(kip1) Knockdown	Down-regulated PTEN and p27(kip1) expression	Apoptosis, senescence phenotype	Human-BMMSCs	25649549
		Regulated protein kinase B (AKT) signaling			
		Enhanced IL-10 and TGF-β and reduced IL-17 and IL-6			
		Increased Treg/Th17 cells			
	Nampt overexpression	Up-regulated intracellular concentrations of NAD+ and SIRT1 expression and activity	Senescence phenotype	Rat-BMMSCs	28125705
	NANOG overexpression	Fortified the actin cytoskeleton and ACTA2	Myogenic differentiation	Human-hair follicle MSCs	28125933
		Restored contractile function			
	Dicer1 overexpression	Increased miR-17 family (miR-17-5p, miR-20a/b, miR-106a/b and miR-93)	Differentiation	Human-BMMSCs	25361944
		Decreased miR-93, miR-20a and p21 expression	Stemness		
	miR-10a overexpression	Repressed the KLF4-Bax/Bcl2 pathway	Apoptosis, survival, differentiation	Human-BMMSCs	29848383
		Activated AKT and stimulated the expression of angiogenic factors	Angiogenesis		
	Lcn2 overexpression	Decreased senescence induced by H2O2	Proliferation, cloning	Human-BMMSCs	24452457
	FGF-21 overexpression	Decreased mitochondrial fusion and increased mitochondrial fission	Senescent phenotype	Human-BMMSCs	31178962
	NDNF overexpression	Activated the AKT signaling	Proliferation	Human-BM/ADMSCs	30062183, 31287219
			Migration		
			Angiogenesis		
	PEDF Knockdown	Induced cellular profile changes	Proliferation	Mouse-BMMSCs	21606086
			Migration		
	MIF overexpression	Activated autophagy	Cell survival after transplantation	Human-BMMSCs	31881006
			Reduced cellular senescence		
			Angiogenesis		
Phamacological	TMP	Inhibited NF-κB signaling	Proliferation, cell cycle	Rat-BMMSCs	31171713
approach		Modulated Ezh2-H3k27me3	Anti-inflammatory and angiogenesis	Mouse-BMMSCs	29488314
	RSV	Regulated SOX2	Multipotency	Rat-BM/ADMSCs	25132403, 26456654, 31440387, 27049278
		Activated SIRT1 expressison	Self-renewal		
		Decreased ERK and GSK-3β phosphorylation and β-catenin activity	Senescence phenotype		
		Promoted insulin secretion of INS-1 cells via Pim-1	Paracrine function		
	Artemisinin	Activated the c-Raf-ERK1/2-p90rsk-CREB pathway	Survival, apoptosis	Rat-BMMSCs	31655619
		Reduced the level of ROS production			
		Enhanced the levels of antioxidant enzymes including SOD, CAT and GPx			
		Increased ERK1/2 phosphorylation			
	Largazole or TSA	Affected histone H3 lysine 9/14 acetylation and histone H3 lysine 4 dimethylation	Proliferation	Human-UCMSCs	23564418
			Osteogenic differentiation		
	CASIN	Reduced Cdc42-GTP	Proliferation, differentiation	Rat-ADMSCs	29804242
		Down-graduated the levels of ROS, p16^*INK4A*^ and F-actin			
		Inhibited the ERK1/2 and JNK signaling pathways			
	DKK1	Hyperactivated the WNT/β-catenin and the p53/p21 pathway	Senescence phenotype	Human-BMMSCs	24130040
	Melatonin	Activated Nrf2 gene through the MT1/MT2 receptor pathway	Survival, senescence phenotype	Canine-ADMSCs	30362962
		Stimulated ERAD, alleviated ERS			
		Inhibited NF-κB pathway			
	SGJ	Promoted lysosomal acidification	Senescence phenotype	Rat-BMMSCs	30526663
		Increased the concentration of H+ and the protein expression of LAMP1/2	Cell morphology		
		Suppressed the expression of p21 and reduced SA-β-gal positive cells	Proliferation		
		Promoted LC3B but reduced the p62/SQSTM1 protein	Autophagy		
	ABT-263/navitoclax	Revealed a senolytic effect	Senescence phenotype	Human-MSCs	29669575
	IDB	Increased the expression of Bcl2, Nanog, octamer-binding transcription factor 4, E-cadherin	Apoptosis	Rat-BMMSCs	29393352
		Decreased the expression of N-cadherin and vimentin	Migration		
		Increased proliferating cell nuclear antigen, cyclinD1 and cyclinD3	Proliferation, cloning		
	Fucoidan	Regulated SMP30 and p21	Proliferation, cell cycle	Human-ADMSCs	29642406
		Regulated CDK2, CDK4, cyclin D1, and cyclin E proteins			
		Regulated FAK-AKT-TWIST signal transduction			
	LC	Decreased the population doubling time	Proliferation, senescence phenotype	Rat-ADMSCs	27943151
	RAPA	Improved immunoregulation	Survival, senescent phenotype	Human-BMMSCs	27048648
		Inhibition of the mTOR signaling pathway			
	Rg1	Decreased the rate of SA-β-gal positive cells	Proliferation	Human-BMMSCs	30055206
	EGCG	Activated Nrf2	Senescence phenotype	Human-BMMSCs	27498709
		Down-regulated the p53/p21 signaling pathway			
	DHJST/Ligusticum chuanxiong	Up-regulated BMP-2 and RUNX2 gene expression	Osteogenic differentiation	Human-BMMSCs	28040510
		Activated of SMAD 1/5/8 and ERK signaling	Senescence phenotype		
	Curcumin	Reduced the population doubling time	Proliferation	Rat-ADMSCs	29017189
	Apocynin	Suppressed NADPH oxidase	Senescence phenotype	Mouse-BMMSCs	26686764
		Reduced p53 expression			
	R-SFN (low doses)	Antioxidant properties	Proliferation, apoptosis, senescence phenotype	Human-BMMSCs	21465338
	1,25-VD3	Decreased systemic phosphate levels	Proliferation, apoptosis	Human-BMMSCs	22242193
Cytokine	MIF	Interacted with CD74	Self-renewal	Rat-BMMSCs	25896286
supplementation		Activated AMPK-FOXO3a signaling pathways	Senescence phenotype		
	IGF1	Activated the IGF1R/PI3K/AKT signaling pathway	Proliferation	Mouse-BMMSCs	31660081
			Stemness		
			Senescence phenotype		
	FGF/FGF-2	Regulated PI3K/AKT-MDM2 pathway	Stemness	Human-BMMSCs	21527526, 17532297
		Inhibited ROS and TGF-β	Proliferation		
	Recombinant human HSP70	Suppressed expression of p16 and p21	Proliferation	Mouse-ADMSCs	27091568
		Induced expression of superoxide dismutase and SIRT-1			
	Jagged1	Activated Notch signaling pathway	Senescence phenotype	Human-BMMSCs	28151468

Pharmacologic approaches and cytokine supplementation have also shown promise for delaying senescence (Table 3). Inhibiting mechanistic target of rapamycin by rapamycin treatment enhanced autophagy and myogenic differentiation in aged stem cells (Takayama et al., 2017), while pretreatment with MIF rejuvenated MSCs in a state of age-induced senescence by interacting with CD74 and thereby activating 5′AMP-activated protein kinase-Forkhead box O3a signaling (Xia et al., 2015). Pharmacologic antagonism of lysophosphatidic acid, a ubiquitous metabolite in membrane phospholipid synthesis, extended the lifespan of MSCs in culture and increased their clonogenic potential while preserving their capacity for both osteogenic and adipogenic differentiation (Kanehira et al., 2012). However, as the effects of these approaches can vary–for instance, fibroblast growth factor supplementation resulted in the loss of osteogenic/adipocytic differentiation potential in long-term cultures (Gharibi and Hughes, 2012)–further research is needed to evaluate their long-term safety and efficacy.

## Conclusion and Future Directions

Mesenchymal stem cells senescence both *in vivo* and *in vitro* can affect MSC characteristics, which has important clinical and safety implications. MSCs must be expanded for several PDs to meet clinical dose requirements, but cellular aging significantly hinders the generation of sufficient numbers of cells. In this review, we discussed the properties of senescent MSCs and the functional changes and cellular mechanisms involved, and highlighted potential rejuvenation strategies. However, the current knowledge of senescence is mainly based on bulk-cell data. Recent technical advances such as single-cell RNA sequencing, extended time-lapse *in vivo* imaging, and genetic lineage tracing will provide a more complete understanding of the MSC aging process, making it possible to slow senescence or even rejuvenate aged MSCs. Additionally, bioinformatics-based analyses of the genome–environment interactions involved in aging can provide potential drug targets for senescence intervention. Given that functional attrition and reduced regenerative potential in stem cells are an important aspect of aging in organisms, MSC rejuvenation holds considerable promise for broadening the applications of MSC-based therapy.

Mesenchymal stem cells-based therapy has several limitations, including the invasive process of collecting the cells and their inherent immunogenicity, as well as the large numbers required to achieve a clinically relevant effect (Berebichez-Fridman and Montero-Olvera, 2018). An increasing number of preclinical trials have reported therapeutic effects exerted by MSC-MVs via paracrine mechanisms in several disease models (Fujita et al., 2018). Whether MSC-MVs are senescent and how senescent MSC-MVs can be identified are outstanding issues to be addressed in future studies.

## Author Contributions

JL and YD searched the literature and drafted part of the manuscript. XL and ZL designed the whole study and revised the manuscript.

## Conflict of Interest

The authors declare that the research was conducted in the absence of any commercial or financial relationships that could be construed as a potential conflict of interest.
